# A Case of Sirenomelia Associated with Hypoplastic Left Heart with a Healthy Co-Twin: A Rare Entity

**DOI:** 10.1155/2018/9361745

**Published:** 2018-06-24

**Authors:** Houda Nasser Al Yaqoubi, Muna Mubarak Al Badi, Farida Mohsin Ambu Saidi, Nasser Shaikhan Taaeeb Al Shafouri

**Affiliations:** ^1^Department of Obstetrics and Gynaecology, Ministry of Health, Ibri Regional Hospital, Ibri, Oman; ^2^Department of Radiology, Ministry of Health, Ibri Regional Hospital, Ibri, Oman; ^3^Department of Paediatrics, Ministry of Health, Ibri Regional Hospital, Ibri, Oman

## Abstract

Sirenomelia is a rare developmental malformation and is incompatible to life. The incidence of sirenomelia, as recorded in the literature, is estimated to be approximately between 1.5 and 4.2 per 1,00,000 births. Around 15% of sirenomelia cases are associated with twin pregnancy, most often in monozygotic cases with an incidence of 7%. In monozygotic twins, the risk of sirenomelia is nearly 100–150 times higher as compared to dizygotic twins or singleton pregnancies. Until now, only two cases of sirenomelia associated with hypoplastic left heart have been reported in the literature. Here, we present a monozygotic twin pregnancy, where one fetus was diagnosed with sirenomelia associated with hypoplastic left heart syndrome and the co-twin was absolutely healthy.

## 1. Introduction

Sirenomelia is a rare developmental malformation and is incompatible to life. It is characterized by a spectrum of fusions in varying degrees of the lower extremities, agenesis or dysgenesis of the kidneys, absent external genitalia, imperforate anus, lumbosacral and pelvic bone anomalies, and a single umbilical artery. Cardiac and central nervous system anomalies have also been reported sporadically [1].

The alternate name of sirenomelia is the mermaid syndrome, as the fused lower extremities in sirenomelia cases resemble mermaids from the Greek and Roman mythology, which were depicted as having the head and upper body of a human and the tail of a fish [2].

The incidence of sirenomelia, as recorded in the literature, is estimated to be approximately between 1.5 and 4.2 per 1,00,000 births. This anomaly is predominantly observed in male fetuses with a male-female ratio of 3 : 1 [3].

Around 15% of sirenomelia cases are associated with twin pregnancy, most often in monozygotic cases with an incidence of 7%. In monozygotic twins, the risk of sirenomelia is nearly 100–150 times higher as compared to dizygotic twins or singleton pregnancies [4].

Until now, only two cases of sirenomelia associated with hypoplastic left heart have been reported in the literature by Sirtori et al. [5] and Turgut et al. [6].

Here, we present a monozygotic twin pregnancy, where one fetus was diagnosed with sirenomelia associated with hypoplastic left heart syndrome and the co-twin was absolutely healthy.

## 2. Case

A 32-year-old, gravida 8, para 5, aborta 2 monochorionic diamniotic twin pregnancy (MCDA) was referred as a high-risk pregnancy to our fetal assessment clinic at 25 weeks of gestation for detailed fetal ultrasonography (USG).

The woman had no history of any past medical illness. All her previous pregnancies had resulted in healthy live births at term by vaginal delivery. This pregnancy had been conceived naturally, and she had not been detected to be hypertensive or diabetic. Furthermore, there was no history of consanguinity with her husband.

The USG revealed an MCDA twin pregnancy with discordant growth (28%) of the twins. One fetus was detected to have left hypoplastic heart, with a single umbilical artery, the absence of the kidney and bladder, and lower extremity deformities with an absence of feet. The other fetus was observed to have a normal growth and biophysical profile. The placentas appeared fused in the posterior uterine wall with barely detectable dividing membranes. The amniotic fluid index was grossly normal for both fetuses. Fetal echocardiography was performed and revealed hypoplastic left heart, transposition of the great arteries (TGA), and pulmonary atresia.

The patient and her husband were informed regarding the poor prognosis of the anomalous twin fetus. At 36 weeks of gestation, after antenatal steroid coverage, an elective caesarean section was performed for the patient in view of the MCDA twin with malposition of the first twin in accordance with the hospital protocol.

**Figure 1:**
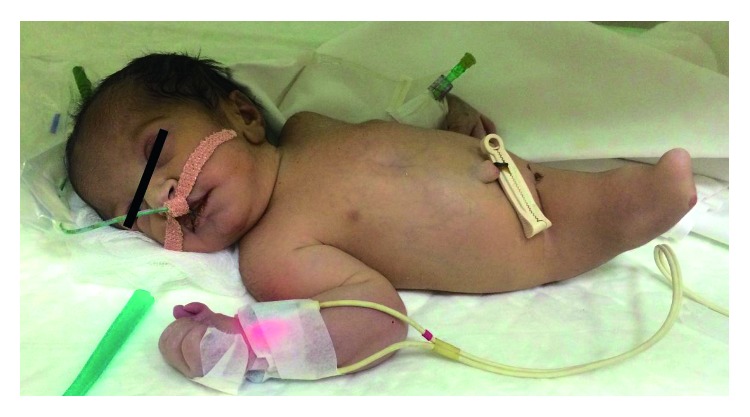
Sirenomelic baby showing no genitalia.

**Figure 2:**
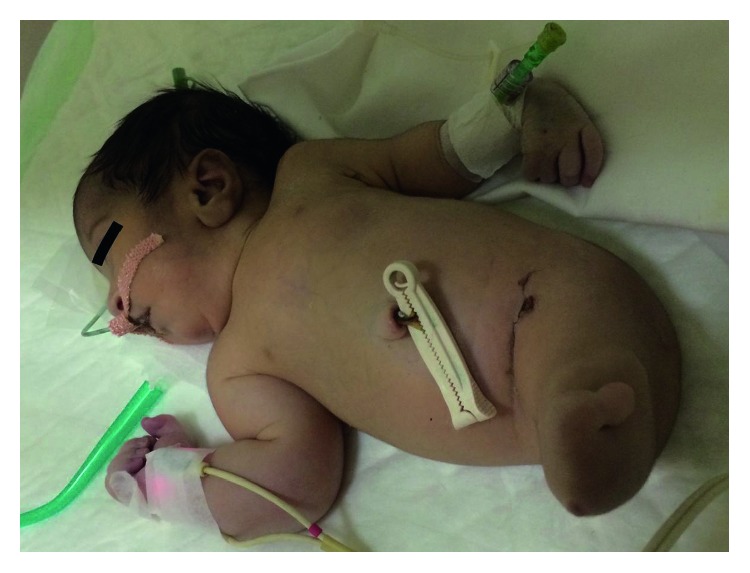
Sirenomelic baby with fused lower extremities and absent feet.

The neonate was born with severe growth restriction with a weight of 1300 gm and cyanosed with a poor Apgar score. As a result, soon after the delivery, it was shifted to the neonatal intensive care unit (NICU). Physical examination of the baby revealed microcephaly (head circumference 29 cm), low-set ears, right hand with polysyndactyly, and complete fusion of the entire lower extremities with an absence of feet (Figures 1 and 2). There was no anal opening, and no external genital organs could be identified. The infant was clinically diagnosed as a case of sirenomelia.

**Figure 3:**
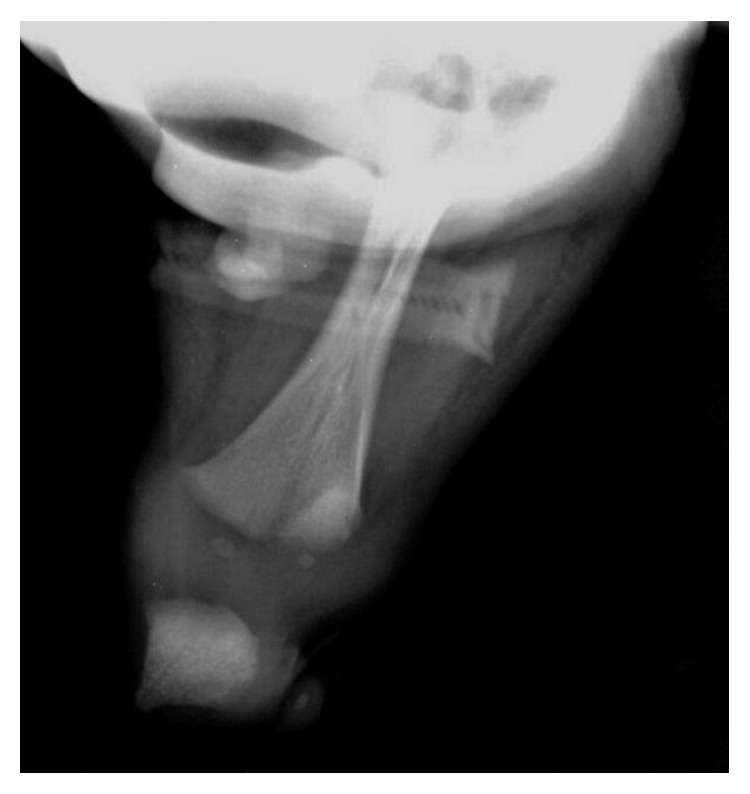
Lower limb X-ray showing complete fusion of both femurs.

Radiologic examinations revealed vertebral deformity at the midthoracic spine, partial sacral agenesis, bilateral hypoplastic iliac bones, complete fusion of both femurs (Figure 3), fused proximal tibia, and an absence of fibula and feet, which was consistent with type VI (sympus apus) sirenomelia [3]. The oesophageal atresia and distended bowel loops present could be secondary to anal atresia.

Neonatal echocardiography was performed, and the diagnosis of hypoplastic left heart syndrome, TGA, and pulmonary atresia was made. USG abdomen and pelvis demonstrated an absence of the bladder and both kidneys with other normal abdominal organs. Chromosomal analysis confirmed the female karyotype 46,XX. The neonate survived for 3 days under human care. An autopsy was not performed due to cultural and social constraints. The other twin was healthy, weighing 2500 gm, and was discharged in good condition after routine preterm care.

## 3. Discussion

The phenotypic characteristics of sirenomelia are associated with varying degrees of severe multiorgan abnormalities commonly involving the urogenital and gastrointestinal systems. Other observed abnormalities in sirenomelia involve lumbosacral and pelvic malformations, which include sacral agenesis, malformed vertebrae and hemivertebrae, absent or malformed external and internal genitalia, imperforate anus, and CNS abnormalities. Pulmonary and cardiac defects are less commonly encountered in sirenomelia [6].

Maternal diabetes mellitus, teratogenic drug or agent exposure, or genetic predisposition is suspected to be the causal factors of sirenomelia. However, the underlying mechanism is still unclear, and many hypotheses have been generated to explain its aetiology. Among the various theories, two important hypotheses, the vascular steal hypothesis and the defective blastogenesis hypothesis, have been described in the literature to explain its pathogenesis. The first theory explains that the abnormal vascular pattern of abdominal and umbilical vasculatures in sirenomelia results in a primary vascular defect that leaves the caudal part of the embryo hypoperfused. The second hypothesis states that, during the blastogenesis of the embryo, damage to the caudal mesoderm leads to overall malformation of the caudal body [2, 6, 7]. Sirenomelia is a typical primary defect of blastogenesis affecting multiple midline primordial during the final stages of gastrulation at the caudal eminence. The caudal eminence is responsible for producing mesenchyme for the lower limb buds and perineum, somites, and vertebrae until the closure of the most caudal neuropore. Lower limb buds arise at stage 13 at somite levels 25–29, from a region produced by the caudal eminence. Interference with the morphogenetic events at the caudal eminence may result in malformations of sacrococcygeal vertebrae, various degrees of caudal dysgenesis, and sirenomelia [8].

During prenatal sonographic diagnosis of sirenomelia, the first sign is progressive oligohydramnios in the second trimester secondary to renal agenesis or abnormalities. The condition is easier to diagnose in the first trimester when the amniotic fluid volume is usually normal and unrelated to the fetal urine production [1, 7].

Surprisingly, in our case, the amniotic fluid volume was normal in both amniotic sacs at 25 weeks of gestation.

In a study involving nine sirenomelia cases, it was observed that five cases had a normal amniotic fluid volume [4].

In another reported case, initially at 18 weeks, marked oligohydramnios was found, but the liquor volume improved week by week. By 25 weeks, an amniotic fluid index of 11.1 cm was seen [9].

In previous studies of sirenomelia in twin pregnancies, expectant management is suggested despite there being a potential risk of preterm delivery or harm to the healthy co-twin in cases of intrauterine death of the affected twin, especially in monozygotic twins. This is owing to the presence of communicating blood vessels between the two fetoplacental circulations [3].

Our case was managed conservatively, and both fetuses were successfully delivered by elective caesarean section at 36 weeks of gestation. The sirenomelic baby survived for 3 days. Although most affected newborns can survive for a few hours, the presence of normal amniotic fluid volume may be the reason of improved respiratory condition that favoured the ex-uterine survival of the infant for few days in our case [10].

Survival of affected infants depends on the degree of the visceral anomalies and the presence of the kidney and renal functions. Death is usually caused by obstructive renal failure secondary to renal agenesis or dysgenesis [11].

There is no robust evidence in the literature pertaining to the association of cardiac anomalies with sirenomelia. In sirenomelia cases, type IV truncus arteriosus is seen commonly but other cardiac abnormalities are encountered sparsely. Till date, only two cases of sirenomelia with hypoplastic left heart syndrome have been reported in the existing literature [5, 6].

In our case, at 26 weeks of gestation, hypoplastic left heart, TGA, and pulmonary atresia were detected by fetal echocardiography, which were confirmed by neonatal echocardiography later on. Ours is the third reported case of sirenomelia association with hypoplastic left heart syndrome.

In this case, the aetiology of sirenomelia remains unclear. There was no history of maternal diabetes or other risk factors, and as the co-twin fetus was healthy, we can exclude environmental factors or teratogenic agent exposure from aetiological factors as both twins would have been equally exposed to the predisposing factors. The causal factor may be associated with genetic aberration in our case. Genetic counselling should be proposed to the parents, as the risk of recurrence is around 3–5% [12].
